# Phakomatoses and Endocrine Gland Tumors: Noteworthy and (Not so) Rare Associations

**DOI:** 10.3389/fendo.2021.678869

**Published:** 2021-05-06

**Authors:** Benjamin Chevalier, Hippolyte Dupuis, Arnaud Jannin, Madleen Lemaitre, Christine Do Cao, Catherine Cardot-Bauters, Stéphanie Espiard, Marie Christine Vantyghem

**Affiliations:** ^1^ Department of Endocrinology, Diabetology and Metabolism, Lille University Hospital, Lille, France; ^2^ University of Lille, Lille, France; ^3^ INSERM U1190, European Genomic Institute for Diabetes, Lille, France

**Keywords:** neurofibromatosis type 1, von Hippel-Lindau, Cowden syndrome, tuberous sclerosis complex, pheochromocytoma, paraganglioma, digestive neuroendocrine tumors

## Abstract

Phakomatoses encompass a group of rare genetic diseases, such as von Hippel-Lindau syndrome (VHL), neurofibromatosis type 1 (NF1), tuberous sclerosis complex (TSC) and Cowden syndrome (CS). These disorders are due to molecular abnormalities on the RAS-PI3K-Akt-mTOR pathway for NF1, TSC and CS, and to hypoxia sensing for VHL. Phakomatoses share some phenotypic traits such as neurological, ophthalmological and cutaneous features. Patients with these diseases are also predisposed to developing multiple endocrine tissue tumors, e.g., pheochromocytomas/paragangliomas are frequent in VHL and NF1. All forms of phakomatoses except CS may be associated with digestive neuroendocrine tumors. More rarely, thyroid cancer and pituitary or parathyroid adenomas have been reported. These susceptibilities are noteworthy, because their occurrence rate, prognosis and management differ slightly from the sporadic forms. The aim of this review is to summarize current knowledge on endocrine glands tumors associated with VHL, NF1, TSC, and CS, especially neuroendocrine tumors and pheochromocytomas/paragangliomas. We particularly detail recent advances concerning prognosis and management, especially parenchyma-sparing surgery and medical targeted therapies such as mTOR, MEK and HIF-2 α inhibitors, which have shown truly encouraging results.

## Introduction

Phakomatoses are a group of systemic diseases linked to ectodermal dysembryogenesis. The term comes from the Greek noun *phakos* (φακός, meaning “lentil” or “spot”) and the word termination *-oma* (for “tumor”), which refer to cutaneous birthmarks, i.e. hamartomas. Other cardinal features involve the central nervous system and the eyes (1). The most frequent forms of phakomatoses are:

- neurofibromatosis type 1 (NF1), also known as von Recklinghausen’s disease,
- von Hippel-Lindau disease (VHL),
- tuberous systemic complex (TSC),
- and Cowden syndrome (CS).

The prevalence is, however, less than 1/2,000 people. These rare diseases were clinically described in the 19th century by famous physicians such as the pathologist Friedrich Daniel von Recklinghausen, and the ophthalmologist Eugen von Hippel, both from Germany, Arvid Lindau, a Swedish pathologist and Désiré-Magloire Bourneville, a French neurologist. Although the hereditary nature of these syndromes was predicted early on, their molecular basis was only elucidated at the end of the 20th century by means of genetic developments and the characterization of the Ras-PI3K-Akt-mTOR pathway in NF1, TSC, and CS, as well as of the hypoxia signaling pathway in VHL.

**Figure 1:**
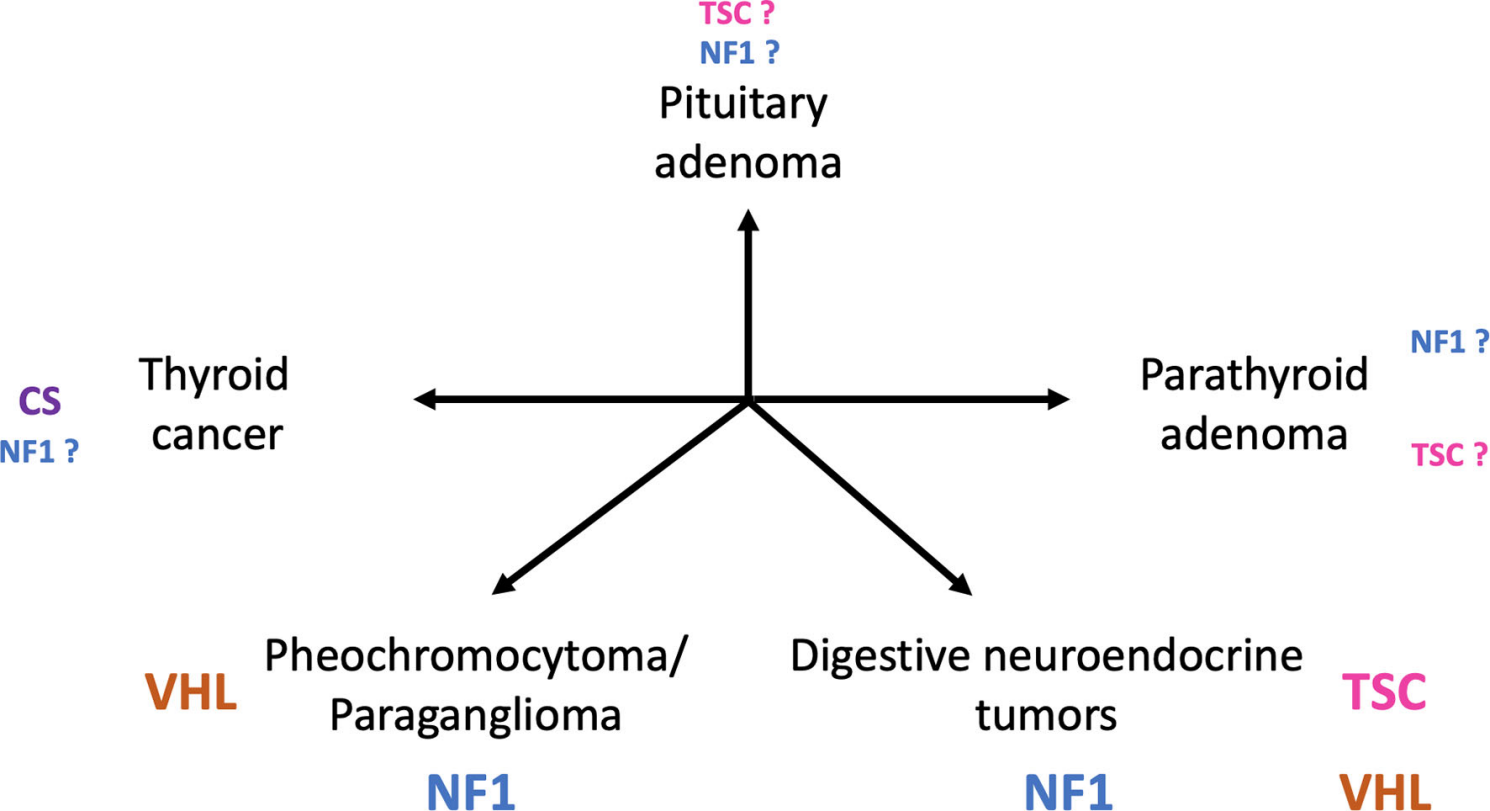
Main tumors of endocrine glands in patients diagnosed with phakomatoses. (TSC, tuberous sclerosis complex; VHL, von Hippel-Lindau disease; NF1, neurofibromatosis type 1; CS, Cowden syndrome).

Besides classical neurological, ophthalmological and cutaneous features, patients with phakomatoses are predisposed to developing tumors of the endocrine glands, with different spectrums for each disease (**Figure 1**).

After a brief review of the pathophysiology of common forms of phakomatoses, we describe the clinical features of endocrine tumors associated with them and focus on their specific features compared with sporadic counterparts; indeed, time of occurrence, clinical expression and prognosis can differ slightly. We then detail recent advances concerning the management of those tumors, especially focusing on parenchyma-sparing surgery in localized disease and pharmacological therapies targeting mTOR, MEK and HIF2-α in advanced/metastatic disease.

## Pathophysiology and Molecular Biology of Phakomatoses

**Figure 2:**
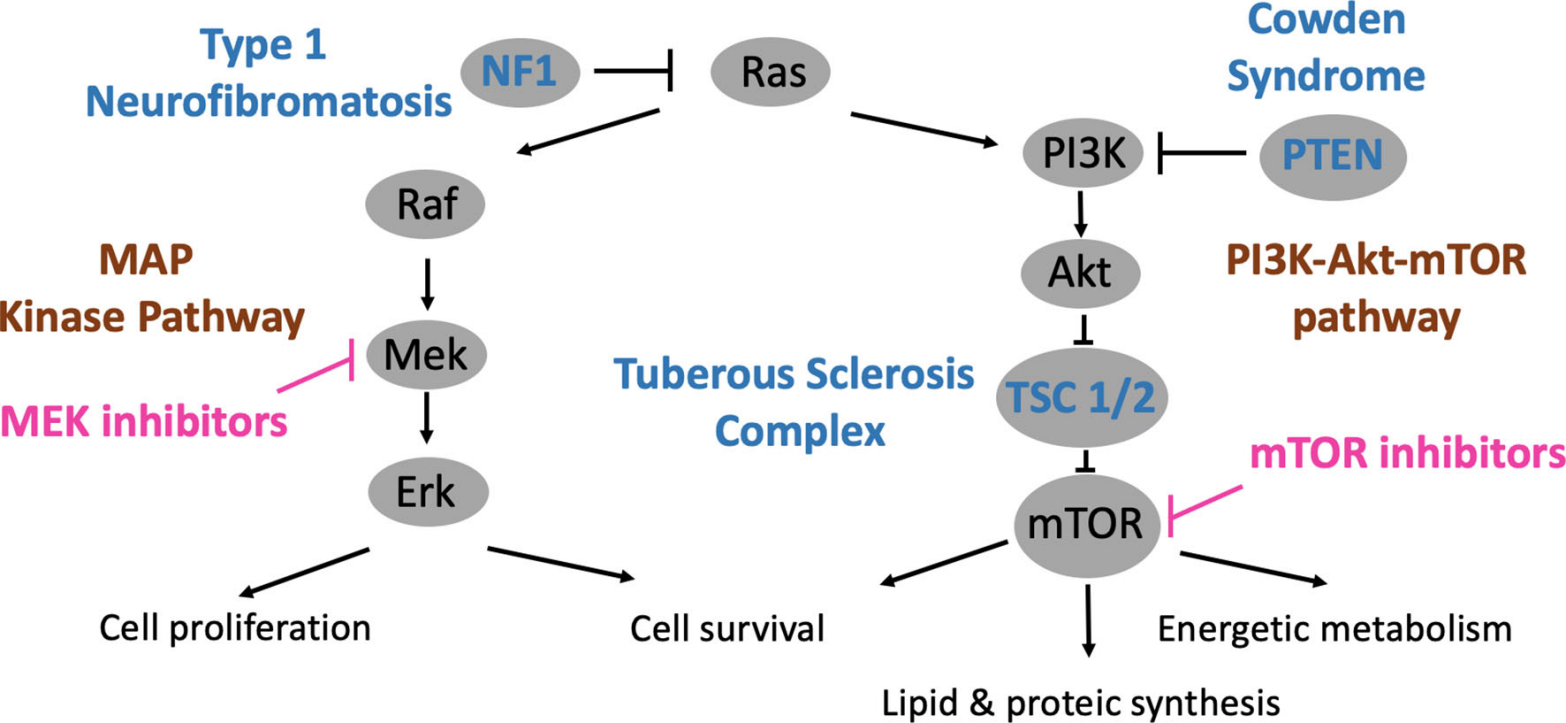
PI3K-Akt-mTOR and MAP kinase pathways, relations with phakomatoses (TSC, NF1 and CS) and therapeutic developments.

NF1, TSC and CS are caused by mutations on genes encoding for different components of the MAP kinase and Ras-PI3K-Akt-mTOR pathways (**Figure 2**). The PI3Kinase-Akt pathway is a classical signaling pathway involved in the regulation of metabolic processes, maintenance of the redox balance, and cell survival and growth (2). PI3Kinase is primarily activated by RAS proteins. This family of proteins (HRAS-NRAS-KRAS) also regulates other signaling pathways such as MAP kinase, which is involved in cell proliferation and survival (3). RAS proteins oscillate between active guanosine triphosphate (GTP)-bound and inactive guanosine diphosphate (GDP)-bound states, and deregulation has been observed in various diseases such as cancer or even psychiatric diseases (4).

Downstream from PI3K and Akt is the mTOR pathway, which ultimately leads to the activation of mTOR complexes 1 and 2 (mTORC1 & 2). The mTOR pathway has been highly conserved during evolution and integrates several environmental cues, such as growth factors, amino acids or glucose, in order to guide cellular growth and fate (5). These multi-protein complexes regulate energy metabolism and lipid/protein synthesis and influence cell survival. In human diseases, mTOR deregulation is involved in cancer, diabetes and ageing (6).

### Neurofibromatosis Type 1

Neurofibromatosis type 1 (NF1), an autosomal dominant disease, is caused by mutations of the *NF1* gene located on chromosome 17q11.2, which encodes neurofibromin (7). This protein accelerates the conversion of active GTP-bound RAS to inactive GDP-RAS. Consistently, MAP kinase and PI3Kinase-Akt-mTOR are deregulated in NF1 (8, 9).

### von Hippel-Lindau Disease

**Figure 3:**
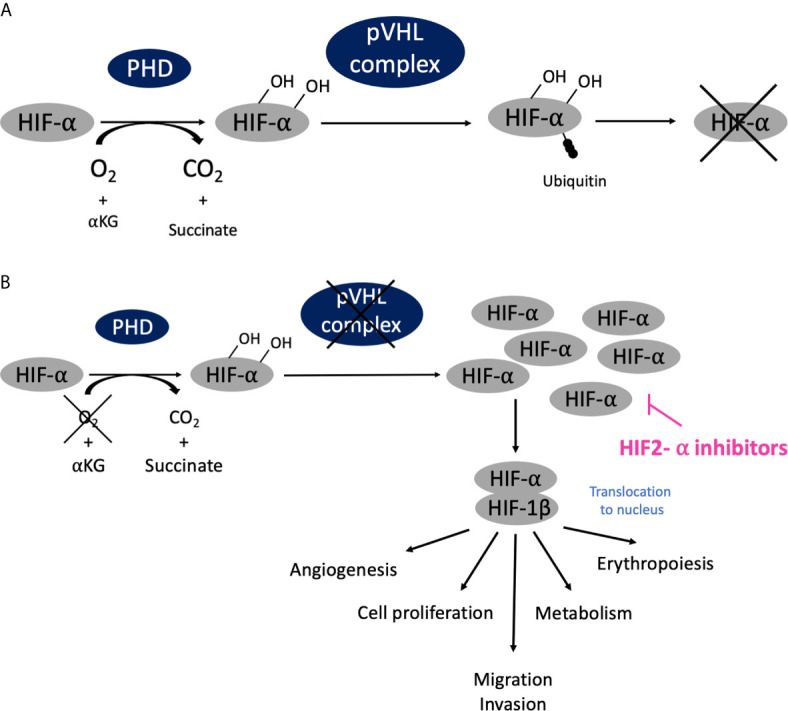
Oxygen sensing pathway, connection with phakomatoses (VHL) and therapeutic developments. **(A)** In normoxic conditions (normal oxygen concentration), HIF-α are hydroxylated on proline residues by prolyl hydroxylase, is then recognized by pVHL complex and ubiquitinated and subsequently degraded *via* the proteasome. **(B)** In hypoxic conditions (low oxygen concentration), HIF-α cannot the be recognized by pVHL and degraded; it accumulates, dimerizes with HIF-1 β, translocates into cellular nucleus and activates transcription of targeted genes. Similarly, when *VHL* is mutated there is also no possibility of HIF-α degradation even in normoxic conditions, and this creates a pseudo-hypoxic state with the HIF-α/HIF-1 β dimer constantly activated, leading to the development of tumor angiogenesis as compensation.

The pathophysiology of another type of phakomatoses, von Hippel-Lindau disease (VHL), is related to a different but major cellular dysfunction: oxygen sensing (10) (**Figure 3**). VHL is an autosomal dominant disease linked to mutations of the *VHL* gene, mapped on chromosome 3p25.3 (11). The VHL protein (pVHL) is part of a multiprotein complex that is also constituted by elongin B and C, Cullin 2 and RBX1, with the whole complex exhibiting E3 ubiquitin ligase properties (12, 13). Under normoxic conditions, hypoxia-inducible factors-α (HIF-α) are hydroxylated on proline residues by prolyl hydroxylase, which allows recognition by pVHL, then ubiquitylation of HIF-2 and subsequent degradation *via* the proteasome (**Figure 3A**). In hypoxic conditions, HIF-α cannot be hydroxylated and recognized by pVHL, and then tend to accumulate, dimerizing with HIF-1 β and activating the transcription of several genes involved in angiogenesis, erythropoiesis, metabolism, cell proliferation, migration and invasion. With *VHL* gene mutations, there is also no possibility of HIF-α degradation even in normoxic conditions, and this create a pseudo-hypoxic state with continuous activity of the HIF-α/HIF-1β heterodimer (**Figure 3B**). Consequently, VHL is crucial in the oxygen sensing process within the body. It also exhibits HIF-independent properties, including assembly and regulation of the extracellular matrix, microtubule stabilization, and regulation of apoptosis.

### Tuberous Sclerosis Complex

Tuberous sclerosis complex (TSC), another autosomal dominant disorder, is related to mutations of the *TSC1* or *TSC2* genes, which are located on chromosomes 9q34 and 16p13.3 (14, 15). These genes encode for hamartin and tuberin, respectively, proteins that have the property of directly inhibiting the mTOR protein. Therefore, mutations on *TSC1* or *TSC2* lead to a hyperactive mTOR pathway.

### Cowden Syndrome

Cowden syndrome (CS), also an autosomal dominant disease, is linked to mutations of the *PTEN* gene, mapped on chromosome 10q23.31 (16). PTEN has the ability to inactivate PI3Kinase, the first kinase that subsequently leads to activation of the mTOR pathway. Consistently, an overactive mTOR pathway is observed when *PTEN* is mutated.

## Main Clinical Features of Phakomatoses

### Neurofibromatosis Type 1

NF1 is the most common form of phakomatoses and affects 1 out of 3000–3500 births worldwide (17). The main symptoms of NF1 are cutaneous, ophthalmological and neurological (**Table 1**). NF1 predisposes to the development of multiple neoplasias including solid cancers, mainly malignant peripheral nerve sheath tumors, as well as malignant hemopathies (18). Endocrine tumors, mainly pheochromocytoma and digestive neuroendocrine tumors, are also observed. Primary hyperparathyroidism, pituitary adenomas and thyroid cancer have been reported but are indeed very rare and, to date, are not considered as classical phakomatoses-associated endocrine tumors (19).

**Table 1:** Neurofibromatosis type 1 (NF1) diagnostic criteria. Adapted from (18).

Clinical criteria	
**Two or more of the following:**	
At least six café-au-lait macules (> 5 mm diameter in pre-pubertal individuals	
and > 15 mm in post-pubertal individuals)	
Freckling in axillary or inguinal regions	
Optic nerve glioma	
At least two Lisch nodules (iris hamartomas)	
At least two neurofibromas of any type, or one plexiform neurofibroma	
A distinctive osseous lesion (sphenoid dysplasia or tibial pseudarthrosis)	
A first-degree relative with NF1	

### von Hippel-Lindau Disease

The classical clinical features of VHL disease usually include CNS and/or retinal hemangioblastoma, endolymphatic tumor, clear cell renal carcinoma, and a predisposition for developing cysts, mainly in the kidney and pancreas (**Table 2**). VHL disease occurs in about 1 per 36,000 births, with a penetrance at 65 years estimated at 90%. The disease is due to a *de novo* mutation in 20% of cases. Mosaic mutations can be identified in a minority (5%) of patients (20, 21). The two main endocrine tumors observed in VHL are pheochromocytoma/paraganglioma (PPGL) and pancreatic neuroendocrine tumors (pNETs). VHL disease is classified into two distinct phenotypes, which are based on the absence (type 1) or the presence (type 2) of PPGL. Type 1 VHL affects 80% of patients and is associated with large deletions or truncating mutations, whereas 20% of patients are in the type 2 group, which is associated with missense mutations. Sub-groups have also been defined within type 2 VHL: type 2a (low risk of renal cancer), type 2b (higher risk of renal cancer) and type 2c (PPGL only).

**Table 2:** von Hippel-Lindau disease diagnostic criteria. Adapted from (10).

**Clinical diagnosis**	
**Family history of VHL and:**	
- CNS hemangioblastoma,	
- or retinal hemangioblastoma,	
- or pheochromocytoma,	
-or clear cell renal carcinoma	
**NO family history of VHL and:**	
- at least 2 hemangioblastomas	
- or at least 2 visceral tumors	
- or one hemangioblastoma AND one visceral tumor	

CNS, central nervous system.

### Tuberous Sclerosis Complex

The classical phenotype of TSC associates cutaneous lesions, neurological features (cortical tubers, subependymal nodules, and subependymal giant cell astrocytomas) and multiple retinal hamartomas (22) (**Table 3**). Its prevalence is estimated at 1/20,000 people. There is no clear genotype-phenotype correlation, but patients with *TSC2* mutations show more severe disease than those with *TSC1* mutations, although it does not involve endocrine tumors. The penetrance is high, with variable expression in the same family. Based on small studies, TSC may be associated with an increased risk of developing digestive neuroendocrine tumors, as well as pituitary and parathyroid adenomas (23).

**Table 3:** Tuberous sclerosis complex diagnostic criteria. Adapted from (22).

**Genetic diagnostic criteria**	
The identification of either a TSC1 or TSC2 pathogenic mutation in DNA from normal tissue is sufficient	
for making a definite diagnosis of tuberous sclerosis complex (TSC).	
**Clinical diagnostic criteria**	
**Major features**	
Hypomelanotic macules (3, at least 5-mm diameter)	
Angiofibromas (3) or fibrous cephalic plaque	
Ungual fibromas (2)	
Shagreen patch	
Multiple retinal hamartomas	
Cortical dysplasia	
Subependymal nodules	
Subependymal giant cell astrocytoma	
Cardiac rhabdomyoma	
Lymphangioleiomyomatosis (LAM)	
Angiomyolipomas	
**Minor features**	
“Confetti” skin lesions	
Dental enamel pits (> 3)	
Intraoral fibromas (2)	
Retinal achromic patch	
Multiple renal cysts	
Nonrenal hamartomas	
**Definite diagnosis: Two major features or one major feature with 2 minor features**	
**Possible diagnosis: Either one major feature or 2 minor features**	

### Cowden Syndrome

The incidence of CS is estimated to be 1/200,000 individuals. Patients with CS classically present with cutaneous features (facial papules, oral mucosal papillomatosis, palmoplantar keratoses), macrocephaly, and Lhermitte-Duclos disease (24) (**Table 4**). They also exhibit a predisposition for developing several types of cancer, the most prevalent being breast, endometrial, renal, colorectal and finally thyroid cancer. Thyroid cancer is typically differentiated, arise from follicular cells and occurs in 20 to 38% of CS patients, with a possible association with promoter or exon 1 mutations (25–28).

**Table 4:** Cowden syndrome diagnostic criteria. Adapted from (24).

**Pathognomonic features**
Adult Lhermitte-Duclos disease (LDD, rare tumor of cerebellum)
Mucocutaneous lesions
Facial trichilemmomas?
Acral keratosis
Papillomatous papules
Mucosal lesions
**Major criteria**
Breast carcinoma
Thyroid carcinoma (non-medullary), especially follicular thyroid carcinoma
Macrocephaly (occipital frontal circumference ≥ 97th percentile)
Endometrial carcinoma
**Minor criteria**
Other thyroid lesions (e.g., adenoma, multinodular goiter)
Intellectual Disability (i.e., IQ ≤ 75)
Gastrointestinal hamartomas
Fibrocystic breast disease
Lipomas
Fibromas
Genitourinary tumors (especially renal cell carcinoma)
Genitourinary malformations
Uterine fibroids
**Operational diagnosis in an individual (any of the following)**
Mucocutaneous lesions alone, if ≥ six facial papules (three of which must be trichilemmomas)
Cutaneous facial papules and oral mucosal papillomatosis
Oral mucosal papillomatosis and acral keratosis
≥ Six palmoplantar keratoses
≥ Two major criteria (one of which must be macrocephaly or LDD)
One major and ≥ three minor criteria
≥ Four minor criteria
**Operational diagnosis in a family where one individual has a diagnosis of Cowden syndrome**
Any one pathognomonic criterion
Any one major criterion and minor criterion
Two minor criteria
Bannayan-Riley-Ruvalcaba syndrome (overgrowth and hamartomatous disorder with multiple subcutaneous lipomas, macrocephaly and hemangiomas)

## Pheochromocytoma/Paraganglioma (PPGL) in Phakomatoses

PPGL arise from adrenal (pheochromocytoma) or extra-adrenal (paraganglioma) chromaffin cells associated with the paravertebral ganglia, and produce one or multiple catecholamines (adrenaline, noradrenaline, dopamine), which results in high blood pressure, palpitations, sweating, headaches, etc. (29, 30). These tumors are usually benign, and malignancy, characterized by distant metastasis, occurs in 8–10% of all PPGL patients: 10% for pheochromocytoma and ~25% for paraganglioma, the difference partially explained by SDH mutations more frequently encountered in paraganglioma (31, 32). Biochemical diagnosis is made with measurements of circulating or urinary catecholamine metabolites (**Table 5**) (33). Half of pheochromocytomas produce significant amounts of adrenaline and are diagnosed by an increase in metanephrines, with a linear relationship between catecholamine concentration and tumor size (34). The other half of pheochromocytomas and extra-adrenal paragangliomas are characterized by a predominant secretion of noradrenaline (35). Predominant production of dopamine is rare, preferentially encountered in head and neck paragangliomas or in malignant PPGLs (36). Some paragangliomas, especially localized in the head and neck, can also be non-secreting.

**Table 5:** General guidelines for diagnosis and treatment of tumors of endocrine glands.

	Biological investigations	Paraclinical investigations	Treatment
**Pheochromocytoma/Paraganglioma**	Plasmatic or urinary catecholamine metabolites (metanephrine, normetanephrine, 3-methoxytyramine)	Imaging: Morphological: CT or MRI, adrenal-specific Functional: ^18^FDG PET-CT or ^18^F-DOPA PET-CT or ^123^I-MIBG scintigraphy or ^68^Ga-SSA PET-CT	Surgery excision Laparoscopy preferred for abdominal location Adrenal-sparing surgery for bilateral PCC
**Gastrointestinal neuroendocrine tumors**	- Chromogranin A - Hormones (gastrin, pancreatic polypeptide, insulin, glucagon, somatostatin, VIP, etc.) - In case of carcinoid syndrome: 24-h urinary 5-HIAA, platelet serotonin	Imaging: -CT injected with contrast agent -Abdominal-pelvic MRI injected with Gadolinium to research metastases -Nuclear imaging: ^68^Ga-SSA PET-CT or ^18^FDG PET-CT or ^18^F-DOPA PET-CT If pancreatic tumor -endoscopy - endoscopic ultrasound to perform biopsies	Anti-secretory treatment: - somatostatin analogue, - telotristat (carcinoid), - PPI (gastrinoma), - diazoxide (insulinoma) Surgical excision Metastatic forms: - surgical excision (hepatic metastasis), - chemotherapy, - targeted therapy (e.g., sunitinib, everolimus), - radiometabolic therapy (^177^Lu-DOTATATE)
**Primary hyperparathyroidism**	- Blood calcium, phosphate, 25-hydroxy vitamin D, parathyroid hormone - Urinary calcium	Imaging: -Morphological: Cervical US and CT -Functional: ^99m^Tc-MIBI scintigraphy, F-choline PET	Surgical excision using minimally invasive cervical surgery
**Pituitary adenoma**	Plasma hormones: cortisol and ACTH at 8 am, 4 pm, 12 am; IGF-1, LH, FSH, estradiol (women); testosterone, SHBG (men); prolactin, TSH, FT4 Dynamic test according to results mainly - 8 am cortisol measurement after 1 mg dexamethasone test at 12 am (if suspicion of hypercortisolism) - OGTT with GH measurement in case of acromegaly suspicion - Intravenous insulin test with GH and cortisol measurement in case of suspicion of hypopituitarism (if no cardiac or neurological impairment)	Imaging: -Morphological: Pituitary MRI -Neuro-ophthalmological examination: e.g., visual fields, Lancaster test	- Somatostatin analogue or dopamine antagonist - Transsphenoidal surgical excision

CT, computed tomography scan; MRI, magnetic resonance imaging; ^18^FDG, ^18^Fluorodeoxyglucose; ^18^F-DOPA, ^18^Fluoro-dihydroxyphenylalanine; ^123^I-MIBG, ^123^I-meta-iodobenzylguanidine; ^68^Ga-SSA, ^68^Ga-somatostatin analogues; PCC, pheochromocytoma; VIP, vasoactive intestinal polypeptide; 5-HIAA, 5-hydroxyindolacetic acid; PPI, proton pump inhibitors; ^177^Lu-DOTATATE, lutetium (^177^Lu) oxodotreotide; US, ultrasound; ^99m^Tc-MIBI, Technetium (^99m^Tc) sestamibi; ACTH, adrenocorticotropic hormone; IGF-1, insulin-like growth factor 1; LH, luteinizing hormone; FSH, follicle-stimulating hormone; SHBG, sex hormone-binding globulin; TSH, thyroid-stimulating hormone; FT4, free thyroxine; GH, growth hormone; OGTT, oral glucose tolerance test.

### NF1

NF1-associated PPGL prevalence varies from 2.9 to 14.6%, with no clear genotype-phenotype association with PPGL risk (37–42). Indeed, in NF1 patients diagnosed with PPGL, the mutational spectrum comprises both intragenic mutations and deletions, with mutations being preferentially located in the cysteine-rich region of the NF1 protein over the RAS-GAP domain (43). The median age at diagnosis is around 40–45 years, which is older than in other genetically-determined PPGLs (44). Most PPGLs are unilateral pheochromocytomas (75%) but bilateral tumors are not rare (up to 17% of cases) and are synchronous in 20 to 40% of cases (38). Paragangliomas are infrequent. In rare cases, mixed tumors can be observed with a ganglioneuroma/ganglioneuroblastoma contingent (45). Gangliocytic paragangliomas can also been observed in ~5% of NF1 patients (46, 47). The catecholamine secretion profile is adrenergic. PPGL tumor size in NF1 (median size 5.8 cm, range 0.8–20 cm) is roughly the same as in sporadic cases. Functional imaging, which is used to investigate bilateral pheochromocytomas and/or paragangliomas and/or metastatic extension, is based on ^18^F-DOPA PET-CT; second choices include ^123^I-MIBG scintigraphy or ^68^Ga-SSA PET-CT (48).

### VHL

PPGL, which defines type 2 VHL, is observed in 20–30% of patients (49–51). The youngest patient diagnosed was 4 years old, and the median age at diagnosis is 25–30 years (52). VHL-associated PPGL are mostly pheochromocytomas; paragangliomas account for only 10 to 20% of these chromaffin tumors. The malignancy potential is lower than in sporadic cases, about 5% vs. 10–17% (53). One-third to one-half of patients with PPGLs have synchronous bilateral pheochromocytomas. The median tumor size is smaller than in the sporadic forms, about 30 mm, and this is possibly related to abdominal screening, which allows early diagnosis (49). Accordingly, the release of catecholamines and the prevalence of associated symptoms and hypertension are lower compared with the sporadic forms (16–55% and 8–46%). VHL-associated PPGL exhibit a specific secretion profile, which is almost exclusively noradrenergic. The absence of adrenaline secretion is due to the epigenetic silencing of phenylethanolamine N-methyltransferase, which catalyzes the production of adrenaline from noradrenaline (54). Functional imaging of PPGL can be performed with various radiopharmaceuticals, which are of particular interest because they can reveal multiple VHL-associated tumors. The recent European Nuclear Medicine Society guidelines prioritized these investigations and, as in NF1 patients, suggested ^18^F-DOPA PET-CT as first-line imaging for exploring potential bilateral pheochromocytomas and/or extra-adrenal paragangliomas (48, 55). If not available or feasible, ^123^I-MIBG scintigraphy or ^68^Ga-SSA PET-CT can be used, with the latter preferred since it also demonstrates great diagnostic performance with regard to neuroendocrine tumors (56).

### Treatment of Phakomatoses-Associated PPGLs

Resection of benign PPGLs should be considered in order to limit cardiovascular complications and prevent unexpected death, which can be triggered with the administration of certain drugs (53, 57–60). Of note is that rare bilateral pheochromocytomas have led to adrenal-sparing surgery (61–63). This allows selective removal of the pheochromocytoma and leaves a sufficient amount of adrenal cortex tissue for maintaining corticosteroid independence and avoiding steroid dependence and its associated comorbidities, i.e. increased mortality risk especially in young patients, and deterioration of quality of life (64, 65). The risk is recurrence of the tumor or the occurrence of acute adrenal crisis in case of stress. Procedures can be performed by an open or preferentially laparoscopic approach, at least for lesions < 50 mm, while larger lesions should be removed by total adrenalectomy (66). Preoperative measures have to be taken, especially the introduction/optimization of anti-hypertensive drugs, in order to limit perioperative-associated morbidity and mortality (67).

Few data are currently available on adrenal-sparing surgery and NF1-associated PPGL, possibly because bilateral pheochromocytomas are present in a minority of patients. However, when performed, the procedure is safe and allows exogenous glucocorticoid-independence, with an estimated risk of recurrence between 0 and 10% (38, 68).

A greater number of studies have been dedicated to VHL-associated pheochromocytomas. One such study considered adrenal-sparing surgery in 26 VHL patients, none of whom developed metastatic pheochromocytoma; 11% exhibited local recurrence that could be treated with a second surgical procedure or active surveillance, 11% presented contralateral pheochromocytoma, and finally 11% became steroid-dependent (69). Similar results were published by an international consortium that compared morbidity and mortality among patients with bilateral pheochromocytomas undergoing total or cortical-sparing adrenal surgery. Of the 184 VHL patients included, 56% had bilateral synchronous pheochromocytomas. Cortical-sparing surgery was successful in 87% of procedures, with a 12% risk of ipsilateral recurrence (70). Sixty percent of VHL patients remained steroid-independent. Therefore, this procedure seems particularly recommended, when feasible, in VHL- and NF1-associated pheochromocytomas, and it can be performed in both adult and pediatric populations (71).

There are currently no specific recommendations for the management of rare cases of advanced/malignant VHL- or NF1-associated PPGL that currently do not differ from sporadic cases. Therapeutic options include locoregional therapies, radionuclide therapy and/or chemotherapy (72, 73). Targeted therapies such as sunitinib have been shown to induce a response in a VHL patient with malignant PPGL (74).

### Screening and Follow-up of NF1 and VHL-Associated PPGLs

#### NF1

**Figure 4:**
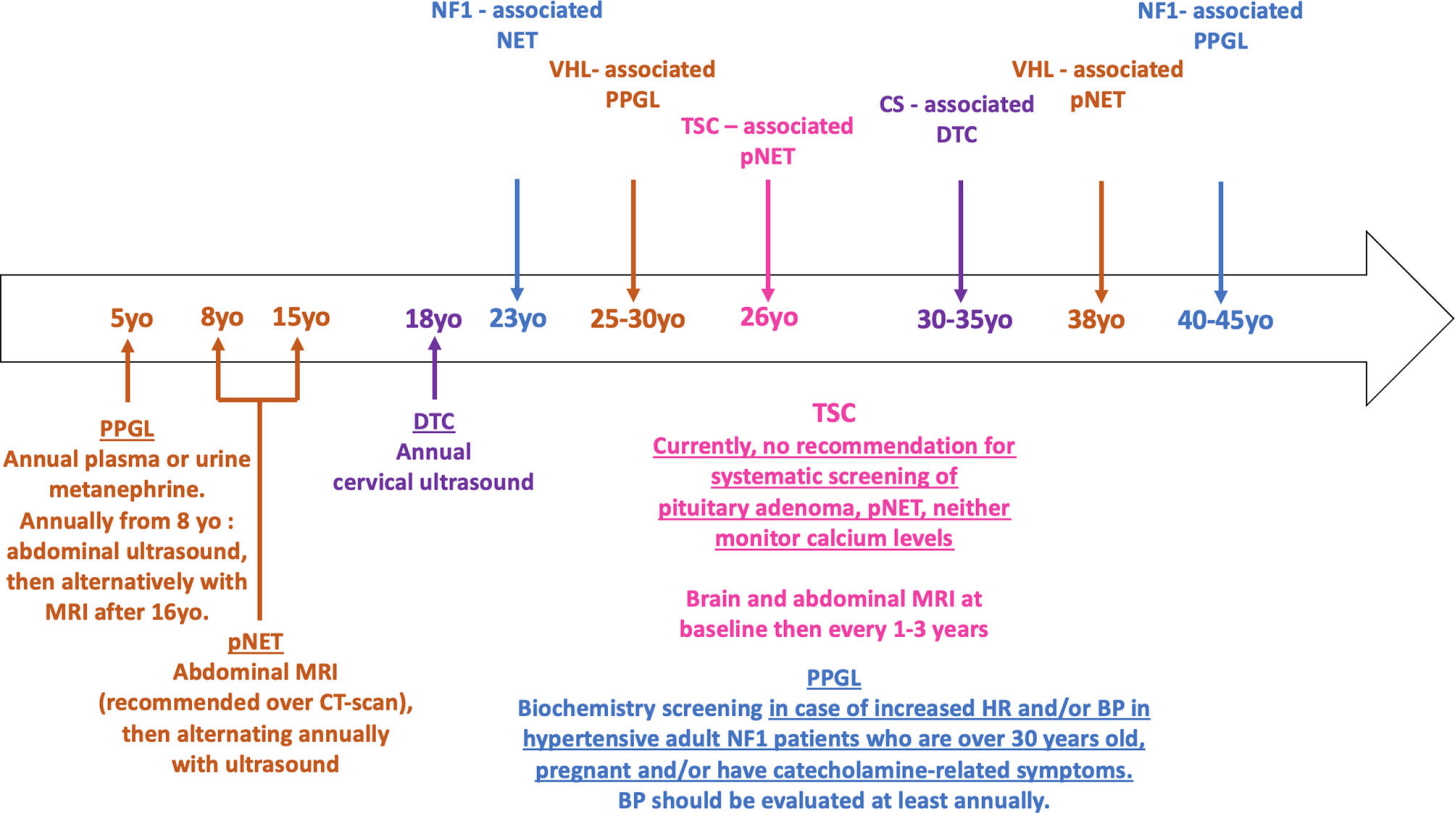
Timeline of phakomatoses-associated endocrine tumors and follow up strategy. The median age of onset of phakomatoses-associated endocrine tumors is shown in the arrow. The various follow up guidelines according to the syndromic presentation are shown below the arrow. **Brown** = VHL; **purple** = CS; **fuchsia** = TSC; **blue** = NF1. HR, heart rate; BP, blood pressure; pNET, pancreatic neuroendocrine tumor; PPGL, pheochromocytoma/paraganglioma; DTC, differentiated thyroid cancer.

There are currently no recommendations for systematic screening of PPGL in NF1 patients in childhood, and experts suggest that investigations for PPGL must be done when there is an increase in heart rate and/or blood pressure (**Figure 4**) (75). Likewise, adult guidelines suggest that PPGL should be considered in hypertensive NF1 patients who are over 30 years old, pregnant and/or have catecholamine-related symptoms (76–79). Blood pressure should be evaluated at least annually, but systematic biochemical or morphological PPGL screening in asymptomatic patients with NF1 is not recommended under the current guidelines. Nevertheless, some studies suggest that only consider screening hypertensive patients would fail to recognize the majority of NF1-associated PPGL (80). Furthermore, prospective morphological and biochemical screening in a French series of 156 NF1 patients found a pheochromocytoma prevalence of 7.7% (12 patients), half (n = 6) of whom were secreting, and with only two of them symptomatic (81). This study and others showing the positive impact of genetic testing on the management and outcome of patients with paraganglioma-pheochromocytoma, and the poor prognosis of PPGL during pregnancy suggest that despite current recommendations, a screening for PPGL, at least before pregnancy, would improve the prognosis of these patients (82–85).

#### VHL

Current Danish and American guidelines as well as French recommendations suggest starting follow-up of VHL patients at a median age of 5 years old, with an annual measurement of plasma or urine metanephrine (**Figure 4**) (86–88). Monitoring of non-invasive imaging can be performed annually from the age of 8 years, initially by ultrasound used first alone, and then alternatively with MRI after the age of 16 years. French guidelines differ slightly and recommend starting abdominal ultrasound at the age of 5 years and the first MRI at 18 years. Functional imaging is useful only when pheochromocytoma is suspected, and it is currently not recommended to repeat it in asymptomatic patients. Particular attention should be paid to screening before pregnancy.

## Digestive Neuroendocrine Neoplasms in PhakomatosEs

Neuroendocrine neoplasms are rare tumors that arise from neuroendocrine cells distributed throughout the entire body (89, 90). Those cells are derived from the embryonic gut (foregut, midgut, hindgut) and can be found in various locations, mainly in the digestive and thoracic regions, with various differentiation and hormonal secretion. Tumor aggressiveness can be assessed according to the WHO grade (grade 1 to 3) based on proliferation index values (Ki-67 and/or mitotic count) (91). Neuroendocrine neoplasms of the gastrointestinal tract comprise neuroendocrine tumors (NETs) and neuroendocrine carcinomas (NECs). Of note, the 2019 WHO classification has modified the cut-off used for the Ki-67 proliferative index to distinguish grade 1 from grade 2 NETs (Ki-67 above or below 3%), emphasized the distinction between grade 3 NETs (low-grade NETs with a high proliferative rate and Ki-67 above 20%) and NECs which are all high grades poorly differentiated aggressive neoplasms (92). Phakomatoses are mainly associated to NETs, which are well differentiated and progress slowly (most of them grade 1) in 85% of cases (93), even if they can be complicated with metastases. Even in metastatic conditions, the median overall survival is typically 5–10 years relative to the location and histological grade in well-differentiated NETs (94–96). In 15% of cases, a functional syndrome (FS) (hypoglycemia, recurrent gastric ulcers, necrolytic migrating erythema) is present, which is related to hormone production (e.g. insulin, gastrin, glucagon). About 4–5% of gastroenteropancreatic tumors arise in the context of an inherited tumor syndrome (mainly MEN1 but also phacomatosis), especially VHL, whereas the incidence of NET is lower in NF1 and TSC. Young age at diagnosis, multiple tumors in multiple organs, and familial history are clinically suggestive of the diagnosis. Neuroendocrine neoplasms in the context of phakomatoses are almost always well-differentiated neuroendocrine tumors. Except in VHL and NF1, tumors themselves do not show specific pathological features (97).

### NF1

NF1-associated NETs are rare, and their prevalence is unknown. Most NF1-associated digestive NETs are located on the ampulla of Vater, followed by the duodenum and pancreas (98–100). The youngest published case was diagnosed at 23 years old and the median age at diagnosis is 48 years, which is 15 years younger than in the general population (94, 101). In a series of 58 NF1-associated NET cases, about 25% (14 cases) were marked with somatostatin antibodies on immunohistochemistry, but only 28% of the patients presented symptoms related to the hormone secretion, i.e. diabetes mellitus, diarrhea, gallstones, and less frequently dyspepsia and hypochlorhydria (102). Gastrinomas and insulinomas have also been observed (103, 104). In NF1, NETs are mostly well-differentiated, rarely multifocal, and can have specific pathological features such as a deceptive tubular/tubuloglandular appearance that can mimic adenocarcinoma. Metastases are present at diagnosis in 14% of reported patients. Specific survival data is not available yet. In a review of 76 published cases, 9% of patients died of NET progression; however, follow-up was short (median 31 months), and the grade as well as median overall survival were unknown. The standard differential diagnosis is gastrointestinal stromal tumors (GISTs), which can also be observed in NF1 patients (105). In this context, the tumors are preferentially located in the ileum/jejunum versus the stomach as in sporadic forms, and most NF1-associated GISTs have a favorable clinical course (106).

### VHL

The VHL pancreatic lesions include solid NETs and cystic lesions. Cystic lesions (simple cysts and serous cystadenomas) are generally asymptomatic and do not require any treatment. They must be differentiated from other cystic tumors that have malignant potential, such as intraductal papillary mucin-producing tumors or mucinous cystic tumors. VHL pancreatic neuroendocrine tumors (pNETs) occur in 11–17% of cases and are frequently multifocal (40%) (107–109). In VHL patients, pNETs present with a characteristic microscopic appearance, with finely vacuolated cytoplasm and lipid-rich cells. Patients with missense mutations (type 2 VHL) rather than truncating mutations or large deletions (type 1 VHL) exhibit a higher prevalence of pNETs, with a hotspot on codons 161/67 in exon 3, which could be associated with a higher risk of metastases although the data are discordant (110, 111). Preferential association with PPGL is also discussed (107, 112). The mean age at diagnosis is 35–38 years; not surprisingly and because VHL does not seem to be involved in hormonal secretion, most patients are asymptomatic with nonfunctional tumors. However, ectopic secretion of ACTH with paraneoplastic Cushing syndrome is possible (113). The pathogenesis of VHL pNETs differ from that of MEN1 or sporadic pNETs notably because of:

- upregulation of genes related to hypoxia-inducible factor molecules, angiogenesis, epithelial mesenchymal transition and/or metastasis, cell cycle and growth factors and receptors (114),
- and hypomethylated CpGs, significantly more common in VHL-related versus sporadic and MEN1-related NETs (115).

The presence of local invasion or locoregional/distant metastasis varies between 7.5 and 12.8%; it reaches 20%, however, for the metastatic forms in the largest cohort, which is lower than in other genetic predisposition syndromes or in sporadic forms (112, 116–118). Metastasis is generally associated with pNETs larger than 28 mm, with a doubling time of 22 months vs. 126 months in non-metastatic lesions (111). Morphological investigations of VHL-associated pNETs do not differ from the management of sporadic forms. In the context of VHL, the differential diagnoses of pNETs include serous cystadenoma or metastatic renal cancer lesions (107). Simple cysts are also frequent (up to 75%), whereas cystic aspects of pNET are uncommon (119). Measurement of plasma chromogranin A can be of interest although there are several limitations of this assay, notably the false-positive results due to hypersecretion of gastrin, which is encountered, among other causes, in case of proton pump inhibitor use, or chronic kidney disease that can be associated with VHL (120). Nuclear medicine imaging recommends ^68^Ga-SSA PET-CT as a first-line investigation, or ^18^FDG PET-CT in more aggressive pNET cases (48). ^18^F-DOPA PET-CT is not useful in this context (55). The progression of pNET lesion size is not linear and may include periods of stability or even apparent decrease in size on imaging (121).

pNET-related mortality is not well documented in VHL patients. In two studies focusing on patients presenting with pNETs, tumor-associated mortality was estimated between 6.9 and 9.5%. However, death was associated with pNETs in 29 to 50% of VHL patients (108, 111). Ten-year overall survival is estimated at 50% in non-operated patients with a tumor size greater than 2.8 cm and rises to 94% in operated patients presenting with a pNET size less than 1.5 cm. No correlation was identified between VHL genotype and mortality.

### TSC

The link between TSC and digestive NETs, especially pancreatic, is recognized because of the pathogenic role of the mTOR pathway in the development of NETs (122). However, the onset of those tumors in TSC remains low, with an estimated incidence of 1%, which can question the causal relation between these two conditions. Nevertheless, since the availability of medical treatment such as everolimus, their incidence could increase with the morphological follow-up of kidney lesions. Genetic data, available only in a small proportion of reported patients, revealed *TSC2* mutations, most of them in or just upstream of the GTPase-activating protein (GAP) domain (exons 33–36) (123). *TSC1* mutations can also be found, however to a lesser extent (124). The largest study focusing on pNETs in TSC patients (n = 18) found an average age of 26 years at diagnosis, significantly younger than in the sporadic cases; so, development in pediatric age or in young adults should be emphasized (123). pNETs were functional in 44% of patients, mostly due to insulin secretion resulting in hypoglycemia (125). The diagnosis can be challenging because of neurological features in TSC patients with seizures; the seizures may be attributed to neurological lesions, while they are indeed related to underrecognized insulinoma-related hypoglycemia, especially in those patients with intellectual disability. The mean pNET size of 5.1 cm was slightly higher than in sporadic cases, with a cystic aspect in one-third of patients without any multifocality, which is in contrast with other genetic NET predisposition syndromes (126). The proportion of synchronous metastasis was 13%, which is lower than in sporadic cases. The progression and specific associated mortality are unknown. Finally other locations such as rectal NET have also been described (127).

### Treatment of Phakomatoses-Associated Digestive NETs

Treatment of NETs includes the management of tumor volume but also, when present, of the functional syndrome. The risk of resection must always be weighed with the risk of diabetes, especially in case of multiple tumors.

#### Functional Syndrome

Treatment of the functional syndrome does not differ from that of sporadic tumors, and we will only consider the most frequent secretions. The functional syndrome can be cured with surgical resection of the NET. However, while awaiting surgery and/or in advanced disease, medical treatment can be initiated in order to limit symptoms that may be life-threatening (128). In case of NF1-associated somatostatin-related symptoms, management is not well defined and can include treatment of diabetes, cholecystectomy, and pancreatic enzyme supplementation. Surprisingly, in a few cases the administration of somatostatin analogues can lead to clinical improvement (128–130), although careful monitoring is needed because hypoglycemia may worsen in some patients (131). Insulinoma-induced hypoglycemia can be reversed with small frequent meals and diazoxide. In malignant insulinoma, everolimus and sunitinib as well as pasireotide have been shown to improve glucose levels (132–134).

#### Antitumor Treatment of NETs

##### NF1

Treatment of NF1-associated NETs does not differ from the management of sporadic cases, whether or not they are metastatic. Treatment of localized NETs is based on tumor resection, which can be performed endoscopically or surgically according to NET location, size and pathological characteristics (128, 135, 136). In metastatic tumors, MEK inhibitors (selumetinib, trametinib) have shown great benefit in NF1 patients because they inhibit the MAP kinase pathway, which is overactivated due to *NF1* mutation. Indeed, a decrease in the size of low-grade gliomas and plexiform neurofibromas has been reported (137–141). However, there is currently no data in NF1-associated NET patients.

##### VHL

Treatment of VHL-associated NETs does not differ from the management of sporadic cases. Given the relatively low risk of malignancy and the high frequency of asymptomatic forms, VHL-associated pNETs should not be removed if less than 15 mm in size and slowly progressing at the regular follow-up, with the rare exception of symptomatic forms (142). In case of non-distant metastatic forms, surgery should be considered for tumors with any of the following characteristics: 1) > 2 cm in the head of the pancreas, 2) > 3 cm in the body/tail of the pancreas, 3) doubling time < 500 days, or 4) in case of metastatic locoregional lymph nodes (143, 144). The recommended surgical procedures are enucleation or pancreaticoduodenectomy for pNETs in the pancreatic head relative to the position from the pancreatic duct (145). The risk of this last surgery is associated with a non-negligible morbidity-mortality rate of about 5%, which is much higher than for pancreatic body/tail pNETs in which enucleation or distal or central pancreatectomy should be performed if possible per laparoscopy. Consequently, total pancreatectomy should be discussed only in the very rare cases of symptomatic multifocal pancreatic tumors that cannot be safely enucleated. Post-pancreatectomy diabetes is a classical complication encountered at 10 years in up to 16% of patients after pancreaticoduodenectomy and in 35% after distal pancreatectomy; therefore, physicians and patients should be aware of this risk before validating surgical indication and educate patients to avoid weight gain (146, 147). Islet autotransplantation, now reimbursed through health insurance in France, could be an interesting option for limiting diabetes-associated morbidity; the procedure carries the risk of tumor occurrence in these genetically-determined diseases, although interesting results have been observed in pancreatic adenocarcinoma in an animal model (148, 149).

There is no difference in medical management between VHL-associated and sporadic metastatic pNETs, although antiangiogenic agents have been especially studied because of the specific deregulated angiogenesis mechanism of the disease (128). Nevertheless, there is not yet a direct comparison between VHL-associated and sporadic forms that evaluates the relative efficacy of these drugs. Sunitinib is approved for the management of pNETs and has shown stability in 5 out of 7 cases of VHL-associated pNETs (150, 151). Another study using multiple antiangiogenic treatments (sunitinib, sorafenib, axitinib, pazopanib) showed a partial response in 4 of the 15 VHL patients with cystic or solid pancreatic tumors (152). In a phase 2 trial, 53% of VHL pancreatic lesions responded to pazopanib (anti-VEGFR 1-3, anti-PDGFR α-β, c-KIT), but most of them were serous cystadenomas (153). Interestingly, pazopanib was also shown to reduce the size of renal lesions and hemangioblastomas.

Since the 2010s, specific HIF2-α inhibitors, which showed great benefits in sporadic clear cell renal cell carcinoma in phase1-2 clinical trials, have been undergoing evaluation alone or in combination with immunotherapy and/or antiangiogenic drugs (154). A phase 2 study using the HIF2-α inhibitor MK-6482 in 61 VHL patients with clear cell renal carcinoma and pNETs showed an objective response rate in 64% of pNET cases, with 4 complete responses. The data are not mature yet, but the 12-month progression-free survival rate was 98.3% (155). Therefore, HIF2-α inhibitors could offer promising prospects for VHL metastatic pNETs or even malignant PPGL.

##### TSC

The management does not differ from the sporadic forms, neither in localized or metastatic disease (128, 156). In advanced disease, mTOR inhibitors such as everolimus, which is used in all NET locations (as second-line treatment), could be of particular interest in TSC patients; however, no studies to date have demonstrated specific efficacy in those patients, except for one case with metastatic pNET and a *TSC2* germline mutation that showed partial response shortly after the introduction of everolimus. Support for this hypothesis may be found in the results of the EXIST trials using everolimus in other features of TSC, with morphological responses observed in renal angiomyolipoma, subependymal giant cell astrocytomas and cutaneous nodules (157, 158).

### Screening and Follow up of Phakomatoses-Associated Digestive NETs

#### NF1

Due to their rarity, there are currently no follow-up recommendations regarding digestive NETs in NF1 patients, and in particular no recommendations to perform and repeat abdominal imaging or endoscopy in asymptomatic patients (**Figure 4**) (76, 77). Nevertheless, visualization of the pancreas is suggested for morphological investigation of neurofibromas, pheochromocytomas, or GISTs.

#### VHL

In VHL patients, current surveillance guidelines regarding pNETs and more largely pancreatic lesions suggest first abdominal imaging between 8 and 15 years of age (**Figure 4**) (86, 87). MRI is recommended over CT scan to limit the consequences of repeated ionizing radiation exposure (159). Due to the non-functional characteristic of most pNETs, there is no indication for systematic biological follow-up.

#### TSC

Finally, there are currently no follow-up recommendations regarding digestive NETs in TSC patients; however, required abdominal imaging for the follow-up of renal angiomyolipoma enables simultaneous visualization of the pancreas and could detect non-functional pNETs at an early stage (**Figure 4**) (160).

## Primary Hyperparathyroidism

### NF1

A few cases of primary hyperparathyroidism occurring in NF1 patients have been published (161). The clinical presentation does not differ from that of sporadic cases, and, notably, the diagnosis is not made at a younger age in NF1 patients. Primary hyperparathyroidism is usually associated with a single adenoma, except for few cases of parathyroid carcinoma (162, 163) (**Table 6**). No recurrence has been observed after surgery. Therefore, a causal relationship between hyperparathyroidism and NF1 cannot be assumed.

**Table 6:** Endocrine tumors and phakomatoses specificities.

	NF1	VHL	TSC
**Pheochromocytoma/Paraglanglioma**	Median age 40–45 years	Median age 25–30 years	
	Bilateral in 75% of cases, synchronous in 20% of cases	Bilateral in 15–40% of cases	
	Malignant forms in 10% of cases	Malignant forms in 5% of cases	
	Might be asymptomatic, which does not prevent adrenal crisis		
**Gastrointestinal neuroendocrine tumors**	Median age 48 years		Median age 26 years
	Location: ampulla of Vater > duodenum > pancreas	Location: pancreas Multifocal in 40% of cases	Location: pancreas
	Secretion: somatostatin (7% of cases), gastrin (5%), insulin (3%)		Secretion: 40% of cases, mostly insulin
	Metastasis in 14% of cases	Metastasis in 15–20% of cases; malignancy associated with tumor diameter above 28 mm	Synchronous metastasis in 13% of cases
	Treatment does not differ. In metastatic forms, possibility of using targeted therapy (e.g., MEK inhibitors)	Surgical treatment for localized forms (enucleation, pancreaticoduodenectomy). Treatment of metastatic forms does not differ from sporadic tumors, possibility of using targeted therapy (e.g., MEK inhibitors, antiangiogenic agents)	
**Primary hyperparathyroidism**	9 cases of single adenoma, 1 case of carcinoma, 1 case of multiple adenomas		Only a few cases
	Median age 52 years		Median age 20 years
**Pituitary adenoma**	20 cases of excess GH, including 15 patients associated with sporadic optic pathway glioma		Rarely associated Secreting (GH, ACTH) or nonfunctional
	Median age 13 years		Median age 35 years

NF1, neurofibromatosis type 1; VHL, von Hippel-Lindau disease; TSC, tuberous sclerosis complex; GH, growth hormone; ACTH, adrenocorticotropic hormone.

### TSC

A few cases of parathyroid adenomas have been described in young TSC patients. The occurrence before 20 years of age argues for a causal role of the *TSC* mutation (23, 164). However, recent advances in understanding the biology and pathogenesis of parathyroid adenomas do not seem to involve the mTOR pathway (165). Furthermore, primary hyperparathyroidism is considered the second most common endocrinopathy after diabetes mellitus (166), raising the hypothesis of a coincidental occurrence. However, TSC mutations could also lead to parathyroid adenoma development in an mTOR-independent pathway. In the literature, TSC patients with primary hyperparathyroidism presented symptoms related to hypercalcemia, with one parathyroid lesion at most.

### Management

There are no specific recommendations for surgery and management of hyperparathyroidism in phakomatoses.

### Screening and Follow up of Phakomatoses-Associated-Primary Hyperparathyroidism

There are currently no recommendations for monitoring calcium levels during the follow-up of NF1 or TSC patients (**Figure 4**).

## Pituitary Adenomas

### NF1

Pituitary adenomas are rarely reported in NF1 patients, although these genetic mutations are known to predispose patients to this condition (**Table 6**) (167–170). Excess growth hormone (GH) has been observed, notably in patients with central precocious puberty, but it seems to be associated with optic pathway tumors (OPT) rather than pituitary somatotroph adenomas (171, 172). Although the mechanism underlying excess GH in NF1 is still unknown, a loss of somatostatinergic inhibition from OPTs with dysregulation of GH secretion is suspected, particularly in tumors close to the hypothalamic and pituitary regions.

### TSC

The association between TSC and pituitary adenomas is still debated. In preclinical models of TSC, such as in an Eker rat with *TSC2* germline mutations, pituitary adenomas were observed in 40–60% of cases, and this was associated with premature death attributed to pituitary hemorrhage (173, 174). However, pituitary adenoma in human TSC patients have rarely been reported (23, 175). As TSC surveillance guidelines suggest repeating a brain MRI every 1 to 3 years because of the risk of astrocytoma, it is unlikely that pituitary adenomas are underdiagnosed (160). When diagnosed, TSC-associated pituitary adenomas are secreting (GH, ACTH) or non-functional tumors, and patients are diagnosed at a relatively young age, before 35 years, compared with sporadic cases.

### Management

There is no rationale for managing phakomatoses and especially TSC-associated pituitary adenomas differently from sporadic cases. It should be noted that in *in vitro* situations, everolimus can lead to a significant decrease in cell viability in TSC; however, no human data are currently available (175).

### Screening and Follow up of Phakomatoses-Associated-Pituitary Adenoma

There are currently no recommendations for screening of pituitary adenomas in asymptomatic NF1 or TSC patients (**Figure 4**).

## Thyroid Tumors

Among cases of phakomatoses, thyroid cancer prevalence is increased in Cowden syndrome. Nevertheless, clinical cases have been reported in NF1.

### NF1

The presence of thyroid disease in NF1 may be linked to different mechanisms including autoimmune thyroiditis, metastasis of another cancer, thyroid neurofibromas, and thyroid cancer. The latter is consistent with knowledge that somatic NF1 mutations have been identified in differentiated and anaplastic thyroid carcinoma (176, 177). In exceptional conditions, it can also be favored by GH secretion from pituitary somatotroph adenomas (168). A population-based study estimated the relative risk of NF1 patients developing thyroid cancer to be 4.9 (178). However, only a few cases have been reported in the literature, so a coincidental association cannot be excluded. Different types of thyroid tumors have been reported, such as well-differentiated papillary cancer or medullary thyroid cancer (179–182). Pathologists must be aware of the presence of NF1 because cancer should not be confused with rare intrathyroid neurofibroma (183, 184). Clinical presentation, notably age at diagnosis, does not seem to differ from the usual presentation. Differentiated thyroid cancer can be found through focal hypermetabolism on ^18^FDG PET-CT that is initially performed to distinguish between neurofibromas and malignant peripheral nerve sheath tumors (185, 186).

### Cowden Syndrome

There is a clear molecular rationale linking Cowden syndrome (CS) and differentiated thyroid cancer because mutations resulting in PI3K/Akt pathway activation are known to be involved in thyroid carcinogenesis and cancer progression (176, 187). The diagnosis can be made during childhood or adolescence in 15% of patients, and the majority of CS patients are diagnosed with thyroid cancer before the age of 40 years. The main histological subtypes are papillary carcinoma (classical and follicular variants) and follicular carcinoma, with a potentially higher prevalence of the latter compared with sporadic forms (14–45% vs. 2%) (188–190). These cancers are often associated with specific pathological features such as multiple adenomatous nodules in the context of lymphocytic thyroiditis (190).

### Management

Management of thyroid cancer in NF1 and CS does not differ from that of sporadic cases and includes surgery with or without complementary iodine treatment (191). There is no increased risk of lymph node extension, so systematic lymph node dissection is not recommended and depends on pre-operative ultrasound findings in individual patients. Since a significant proportion of patients with CS also develop nonmalignant thyroid diseases, some authors have suggested prophylactic thyroidectomy (192, 193). However, this procedure is not recommended given the good prognosis of CS-associated thyroid cancer, similar to that of sporadic cases. Recently, a pilot study investigating the benefits of sirolimus, an mTOR inhibitor, in patients with germline *PTEN* mutation demonstrated improvement in skin and gastrointestinal lesions, but there are no data focusing on thyroid cancer (194).

### Screening and Follow up of Phakomatoses-Associated Thyroid Cancer

The European guidelines for CS follow-up suggest an systematic surveillance for thyroid cancer by cervical spine ultrasound (**Figure 4**) (195). An annual investigation starting at 18 years of age is proposed, although the levels of evidence supporting those modalities are moderate and some authors suggest beginning surveillance at 10 years of age (196). Indeed, systematic annual ultrasound monitoring could lead to overdiagnosis and excessive thyroidectomy, therefore follow up frequency could be modified based on the first cervical screening results.

There are currently no recommendations for screening of thyroid cancer in asymptomatic NF1 patients.

## Conclusions

Phacomatoses are a group of rare diseases that can be associated with neoplasia of the endocrine glands. Clinicians must be aware of these features. The main tumors are PPGL and digestive, especially pancreatic neuroendocrine tumors. They can occur at a younger age compared with sporadic cases and are more frequently multiple. Usually benign, they can, however, be aggressive, and surveillance guidelines are available for detecting tumors at early stages and limiting associated morbidity and mortality, especially in VHL. Screening for catecholamine secretion in NF1 appears beneficial according to recent data, since these frequently asymptomatic but life-threatening tumors pose a high risk for cardiovascular morbidity and mortality and have a poor maternal-fetal prognosis in case of pregnancy. All secreting pheochromocytomas should be operated, but parenchyma-sparing surgery must be favored to avoid complete adrenal insufficiency in selected cases with bilateral pheochromocytomas. Management of pancreatic NET depends on the size, number, secretion, aggressiveness (Ki-67) and extra-pancreatic extension of the lesions. The treatment (including surveillance) must be discussed in order to limit surgical risk and post-pancreatectomy diabetes. In advanced/metastatic diseases, besides standard treatments, specific therapies that target the underlying genetic abnormality are under investigation. Importantly, those rare patients should be managed and followed by specialized and multidisciplinary teams and networks to weigh the benefit-risk ratio of each therapeutic strategy.

## Author Contributions

BC and HD wrote the manuscript and are co first authors. AJ, ML, CD, CC-B, and SE contributed to the enrichment and the editing of the manuscript by their comments and remarks. MV supervised the work, wrote and edited the manuscript. All authors contributed to the article and approved the submitted version.

## Conflict of Interest

The authors declare that the research was conducted in the absence of any commercial or financial relationships that could be construed as a potential conflict of interest.

## Abbreviations

ACTH, adrenocorticotropic hormone; CNS, central nervous system; CS, Cowden syndrome; CT, computerized tomography; ^18^FDG, ^18^fluorodeoxyglucose; ^18^F-DOPA, ^18^fluoro-dihydroxyphenylalanine; FS, functional syndrome; ^68^Ga-SSA, ^68^Ga-somatostatin analogues; GH, growth hormone; HIF, hypoxia-inducible factor; ^123^I-MIBG, ^123^I-metaiodobenzylguanidine; APK, mitogen-activated protein kinase; MEN1, multiple endocrine neoplasia type 1; MRI, magnetic resonance imaging; mTOR, mechanistic target of rapamycin; NET, neuroendocrine tumor; NF1, neurofibromatosis type 1; PET-CT, positron emission tomography-computerized tomography; PDGFR α-β, platelet-derived growth factor receptor α-β; PI3K, phosphoinositide 3-kinases; PPGL, pheochromocytoma/paraganglioma; TSC, tuberous sclerosis complex; VEGFR 1-3, vascular endothelial growth factor receptor 1-3; VHL, von Hippel-Lindau disease.
